# Environmental and genetic effects on phytochemical and nutritional composition of onion (*Allium cepa* L.) varieties in South Korea

**DOI:** 10.3389/fpls.2025.1649912

**Published:** 2025-08-15

**Authors:** Muhammad Imran, Hajeong Kang, Eun-Ha Kim, Sang-Gu Lee, Hyun-Min Park, Hanyoung Choi, Seong-Hoon Kim, Seonwoo Lee, Seonwoo Oh

**Affiliations:** ^1^ Department of Applied Biosciences, Kyungpook National University, Daegu, Republic of Korea; ^2^ Biosafety Division, National Institute of Agriculture Science, Rural Development Administration, Jeonju, Republic of Korea; ^3^ National Agrobiodiversity Center, National Institute of Agriculture Science, Rural Development Administration, Jeonju, Republic of Korea; ^4^ Department of Statistics, Dongguk University, Seoul, Republic of Korea

**Keywords:** onion (*Allium cepa*), antioxidant activity, phytochemical composition, genotype × environment interaction, soil nutrients, environmental adaptation

## Abstract

Onions (*Allium cepa* L.) are widely recognized for their antioxidant properties, bioactive compounds such as flavonoids, organosulfur compounds (OSCs), and phenolics. This study evaluate the antioxidant activities, phytochemical composition, and nutrient variations of two onion varieties, Katamaru (KM) and Sunpower (SP), cultivated in two different locations, Muan and Changnyeong, Korea. The antioxidant activity was assessed through enzymatic and non-enzymatic parameters, including peroxidase (POD), polyphenol oxidase (PPO), ascorbate peroxidase (APX), glutathione reductase (GSH), catalase (CAT), superoxide dismutase (SOD), DPPH radical scavenging activity, and total phenolic content (TPC). The results indicated that KM consistently exhibited higher antioxidant potential than SP, with Muan-grown samples demonstrating superior enzymatic and non-enzymatic activities compared to Changnyeong. Where Muan’s alkaline soil and higher phosphorus content favored oxidative stress mitigation. Furthermore, nutrient analysis revealed significant genotype x environment interactions, affecting macronutrient (K, P, Ca, Mg, S) and micronutrient (Na, Cu, Fe, Mn, Zn) accumulation. Additionally, KM demonstrated higher amino acid composition compared to SP in both location, Muan and Changnyeong, likely influenced by environmental factors such as temperature and soil pH. The findings of this study offer insights for improving onion antioxidant and nutrient traits. These results emphasize the role of environmental adaptation and varietal selection in maximizing onion quality and potential health benefits.

## 1 Introduction

Onion (*Allium cepa* L.) is a widely consumed vegetable available in a variety of colors and has become a staple in diets worldwide. Known for its rich nutrient profile, onions are consumed both fresh and dried, significantly contributing to a balanced diet. Due to their nutritional, medicinal, and functional properties, onion production has been steadily increasing, positioning it as one of the most cultivated vegetable crops globally. Today, onions rank as the second most extensively grown vegetable worldwide, following tomatoes (Stoica et al., 2023). They thrive in a range of climates, from temperate to tropical regions. Over the past decade, global onion production has surged, reaching over 98 million tons, with a reported output of 106.59 million tons in 2021, according to FAOSTAT (FAO, 2021). Leading producers include India (26.64 million tons), China (24.16 million tons), Egypt (3.312 million tons), the United States (3.10 million tons), Türkiye (2.50 million tons), and Pakistan (2.30 million tons) (Mehta, 2017). Onions are commonly found in red, yellow, and white varieties in the food market. Although a biennial crop by nature, onions are typically cultivated as annuals to harvest the bulb by the end of the first growing season. The onion bulb comprises a short, disc-like stem (basal plate) and leaf bases arranged in a cylindrical form called scales, which store water and photosynthetic products essential for bulb formation.

Onion processing generates substantial waste, particularly from skins, which can account for up to 60% of total processing waste (Sagar et al., 2022). Despite this, onion skins are often underutilized in products like fertilizers or animal feed, due to strong odor or risks of contamination by white rot (Sclerotium cepivorum) during cultivation. Onion flavor and pungency, resulting from sulfur compounds like allyl propyl disulfides, vary based on intended use: moderately pungent onions are preferred for cooking and seasoning, while highly pungent types are used in sauces and canned soup extracts (Loredana et al., 2019). Research highlights onions’ health benefits, with animal studies and clinical trials indicating potential roles in managing asthma, cancer, diabetes, hypercholesterolemia, and osteoporosis (Min et al., 2015; Marrelli et al., 2018; Sagar et al., 2022). Onion skins, in particular, have been linked to anti-asthmatic, anticancer, and hypocholesterolemic effects, with added benefits for cardiovascular health (Sagar et al., 2022). These effects are primarily attributed to quercetin, a potent antioxidant. Consequently, onions and their by-products hold promise as functional food ingredients, serving as natural antioxidants, preservatives, and additives. Onions are rich in a variety of bioactive compounds, including fructooligosaccharides (FOS), flavonoids, ascorbic acid, and organosulfur compounds (OSCs). These compounds have been associated with numerous health benefits (Sagar et al., 2022). Onions are particularly abundant in OSCs and flavonoids, both known for their antioxidant properties. The total thiosulfinates content can vary across different Korean onion cultivars, which found that red, yellow, and white onions contain 0.20, 0.35, and 0.14 μmol/g FW of thiosulfinates (Choi and Surh, 2014). Thiosulfinates can convert into a variety of sulfur-containing compounds, depending on the reaction conditions. These include diallyl sulfides, vinyldithiins, and ajoenes, among others. Chemically, the OSCs in onions are primarily composed of diallyl monosulfide (DMS), diallyl disulfide (DADS), diallyl trisulfide (DATS), and diallyl tetrasulfide (DTTS) (Pareek et al., 2017). Onion is an excellent source of natural antioxidants. Numerous studies have been conducted to assess the antioxidant properties of onion, revealing that it exhibits significant antioxidant activity. This has been demonstrated through various *in vitro* assays, including ABTS (2,2′-azino-bis-(3-ethylbenzothiazoline-6-sulfonic acid)), DPPH (1,1-diphenyl-2-picryl-hydrazyl), FRAP (ferric reducing antioxidant power), lipid peroxidation, ORAC (oxygen radical absorbance capacity), TAC (total antioxidant capacity), and TEAC (trolox equivalent antioxidant capacity) (Kim et al., 2022). Several factors have been identified that influence the antioxidant activity of onions, such as genetic background, cultivation methods, storage conditions, specific onion parts, extraction techniques, and processing technologies, and can vary among different onion cultivars or varieties, likely due to differences in their genetic makeup (Kim et al., 2024). In Korea, onion cultivation spans a variety of regions, including open fields, gardens, and protected environments such as greenhouses. In 2021, Korea produced approximately 218,500 tons of onions, generating substantial agro-food waste, primarily from onion by-products (FAO, 2021; Canton, 2021). Although precise records on the introduction of onions to Korea are limited, historical references suggest their presence as early as 1908, as indicated in the 1908 Korean Jung-Ang Newsletter’s Horticulture Model Book and Seedling Results. The first large-scale cultivation was initiated around 1930 in Muan, Jeollanam-do, led by figures such as Jang Eui-jin and Kang Dae-gwang. Since then, the Jeollanam-do region, particularly counties like Muan, Hampyeong, Jangseong, Naju, and Yeonggwang, has become a major hub for onion farming (Park, 2012). In Changnyeong, onion farming began in the 1940s when locals imported ‘Jusuhong’ seeds from Japan to develop onions as a cash crop (NSRC, 2019). Therefore, the current study was aim to validate the comparative phytochemical analysis of different onion varieties under different climatic condition.

## 2 Materials and methods

### 2.1 Sample collection and preparation

For the current study the two onion varieties sunpower [(SP) early maturing variety)] and Katamaru [(KM) medium maturing variety)] was provided and grown at two location, Muan (Bioenergy Crop Research Institute, National Institute of Crop Science, RDA, Muan 58545, Korea) and Changyeong (Onion Research Institute of Gyeongsangnamdo Agricultural Research and Extension Services, Changyeong 50319, Korea). During the growing seasons two onion varieties sunpower (SP) and Katamaru (KM) were cultivated at mid-November (2023/11/16) and harvested base on their maturity (SP harvested at 17 May 2024, and KM harvested 22 June 2024). The climatic condition of each site is presented in **Supplementary Table S1**.

The plots used in the study were first tilled twice (7-day intervals) and were then exposed twice to weed and vegetation control the week before seed sowing for each location. The plots at each site were arranged in a balanced strip pattern, with two rows measuring 10m each for each variety, having 50 plants in each row with a 30cm distance between each row and a 20cm spacing between each plant. The management of cultivation followed a uniform standard across all locations to ensure comparability. Fertilization was applied at sowing with a recommended dose of nitrogen (16 kg/1000 m2), phosphorus (15 kg/1000 m2), and potassium (10 kg/1000 m2), as per the guidelines of the Rural Development Administration (RDA), South Korea. Post-emergence fertilization (urea, 16 kg/1000 m2) was conducted at February 2024, and irrigation was applied weekly during dry spells to maintain optimal soil moisture. Pest and disease control measures were performed using chemical-free practices, relying on manual monitoring and removal of affected plants to preserve environmental sustainability (Kim et al., 2021). The soil samples for each site were collected and analyzed according to the reported method of (Sparks, 1996) (**Supplementary Table S2**). A 5 replicates were randomly selection from the obtain onion sample and where bring to Biosafety Division, National Institute of Agriculture Science, RDA Jeonju, and the sample peel were remove and cut into small pieces and immediately put in liquid nitrogen, afterward the samples were put in mash bag and subjected to freeze dry for two week. After completing the freeze dry the sample where grounded to fine powder and keep in -80 °C until used. Moreover, 5 replicates were used to measure onion shape index, using vernier caliper (Shape index= height/dimeter), and values were compared as 1 indicates a spherical bulb, >1 indicates an elongated bulb, and<1 indicates a flattened bulb.

### 2.2 Quantification of antioxidant activities

#### 2.2.1 Peroxidase and polyphenol oxidase activity

POD levels were determined using the method described by (Imran et al., 2022). Briefly, 0.2 g of fresh onion samples were grounded in 5 mL of 0.1 M phosphate buffer (pH 6.8), and the obtained 50 µL of supernatant was added to a reaction mixture containing 25 mL of 5% H_2_O_2_ and 100 mL of 100 mM phosphate buffer (pH 5.5) followed by 50 mL of 50 mM pyrogallol. Phosphate buffer (pH 5.5) was used as a negative control in the absence of the enzyme. POD activity was determined as a unit change per minute by measuring the absorbance at 470 nm for 3 min.

#### 2.2.2 Reduced glutathione activity

The reduced glutathione (GSH) and CAT activities were quantified using a method described earlier (Imran et al., 2022; Kim et al., 2022). Briefly, 0.2 g of fresh onion samples were homogenized in 5mL of 5% TCA, 100 μL of the supernatant was transferred to a 96-well plate, and 50mL of Ellman’s reagent was added, followed by 100mL of 150mM phosphate buffer. The absorbance was recorded at 420 nm with three replicates.

#### 2.2.3 Ascorbate peroxidase activity

For APX activity, 100 mg of the fresh onion sample was extracted with 1mL of 50mM phosphate buffer (pH 7.0) containing 1mM of ascorbic acid and 1mM of EDTA. After homogenization, the mixture was centrifuged at 4,830×g for 15 min at 4°C, 200mL of the supernatant was taken, and absorbance was recorded at 290nm as described previously by (Halo et al., 2015; Imran et al., 2022).

#### 2.2.4 Superoxide dismutase activity

For quantification of SOD, the method of McCord and Fridovich (1969) (McCord and Fridovich, 1969) was used with modifications. Briefly, 0.2 g of the fresh onion sample was extracted with 5 mL of SOD extraction buffer (50 mM of Tris–HCl and 10 mM of EDTA), and 50 mL of the supernatant was mixed with 150 μL of extraction buffer followed by 50 mL of pyrogallol in a test well (A). Fifty microliters of the supernatant was added to 200 mL of the extraction buffer in a test well plate (B), and 150 mL of the extraction buffer followed by 100 mL of pyrogallol was added in the test well (C). The SOD absorbance was recorded at 420 nm. SOD activity (%) was calculated using the following formulas:


SOD activity (%) =[(1-(A-B/C)] ×100,

where (A) is reading 1, (B) is reading 2 and (C) is reading 3.

#### 2.2.5 Catalase

For quantification of CAT, the method of (Halo et al., 2015; Imran et al., 2022) wasfollowed. Briefly, Catalase activity was measured using a modified method. Briefly, 200 mg of the fresh onion t sample was homogenized with 5 mL of extraction buffer. To prepare 250 mL of buffer, 1.5 g of Tris–HCl, 0.152 g of MgCl_2_, and 0.081 g of EDTA were dissolved in distilled water, stirred continuously until fully dissolved. Separately, 0.25 g of polyvinylpyrrolidone (PVP) was dissolved in 30 mL of distilled water on a magnetic stirrer at 35°C for 20 minutes, then filtered through vacuum filtration. The PVP solution was added to the rest of the extraction buffer. After extraction, the sample was vortexed and centrifuged at 10,000 rpm for 10 minutes. The supernatant was transferred to a new tube, and 50 µL of the supernatant was mixed with 150 µL of 10 mM phosphate buffer and 50 µL of 0.2 M H_2_O_2_. The absorbance was read at 240 nm, 255 nm, and 280 nm to determine CAT activity.

#### 2.2.6 DPPH (2,2-Diphenyl-1-picrylhydrazyl) activity

For quantification of DPPH, the method of (Blois, 1958; Imran et al., 2022) was followed. Briefly, the DPPH assay was performed to assess antioxidant activity, following a modified protocol. Approximately 300 mg of the plant sample was homogenized with 5 mL of 80% ethanol, vortexed, and sonicated for 30 minutes. The sample was then centrifuged at 10,000 rpm for 15 minutes, and the supernatant was collected. To perform the assay, three sets of wells were prepared as follows: Blank: 250 µL of ethanol only, DPPH Control: 100 µL of 80% ethanol and 100 µL of 0.5 mM DPPH, and Sample: 25 µL of supernatant, 50 µL of 80% ethanol, 100 µL of 0.5 mM DPPH, and 100 µL of 100 mM acetate buffer. The samples were incubated at room temperature for 20 minutes, and absorbance was measured at 517 nm. DPPH activity was calculated using the formula:


Calculation Formula:DPPH%=(100-(Sample-Blank)×100/DPPH)

#### 2.2.7 Total polyphenol content and total flavonoid content

For quantification of TPC, the method of (Singleton et al., 1999; Imran et al., 2022) wasfollowed. Briefly, Total polyphenol content was estimated following an ethanol extraction method. A 300 mg plant sample was homogenized with 5 mL of 80% ethanol, vortexed, and sonicated for 30 minutes. The mixture was then filtered under vacuum through a glass filter, and an additional 5 mL of 80% ethanol was added to bring the final volume to 10 mL. From the filtered extract, 1 mL was transferred to a new tube and mixed with 1 mL of Folin-Ciocalteau reagent and 1 mL of 10% Na_2_CO_3_. This solution was incubated at room temperature for 1 hour, followed by centrifugation at 10,000 rpm for 15 minutes. Finally, 250 µL of the supernatant was transferred to a spectrophotometer cuvette, and absorbance was recorded at 740 nm. Total flavonoid content (TFC) was measured following the method used by Woisky and Salatino (Woisky and Salatino, 1998; Joković et al., 2024). Samples were prepared by mixing 600 µL of the extract solution with 2580 µL of a reaction mixture (80% C2H5OH, 10% Al(NO3)3 × 9 H2O, and 1 M C2H3KO2). The samples were then incubated at room temperature for 40 min and the absorbance was measured at 415 nm. The quercetin (Qu) calibration curve (10–100 mg/L) was used for the calculation of total flavonoid concentration, which was expressed as quercetin equivalents per g of dry weight of extract (Qu/g DW). All measurements were performed in triplicate.

### 2.3 Quantification of proximate components

All proximate components were analyzed following the standard protocols set by the Korea Ministry of Food and Drug Safety (MFDS). Moisture content was assessed using the specified procedures from prior studies (Kim et al., 2021), Crude fat content was determined in accordance with the method described in (MFDS, 2019e) while crude protein content was calculated based on total nitrogen using the Kjeldahl method (MFDS, 2019e). Ash content was measured following the approach outlined in (MFDS, 2019a). Carbohydrate content was estimated as 100% minus the total percentages of protein, lipid, ash, and moisture. Crude fiber content was analyzed based on the Association of Official Analytical Chemists method 962.09 (MFDS, 2019b; Kim et al., 2021). Acid detergent fiber (ADF) and neutral detergent fiber (NDF) contents, enzymatic-gravimetric methods were applied, in line with the MFDS food code (MFDS, 2019d; Kim et al., 2021).

### 2.4 Quantification of amino acids

The method of (Park et al., 2015; Lee et al., 2017) was employed to identify phytosterol and Policosanols. A 0.2g sample was mixed with 5a-cholestane as an internal standard (IS). The lyophilized samples were derivatized in a thermomixer by adding 30µl of MSTFA and 30µl of pyridine, followed by 30 minutes of shaking incubation at 60°C (Eppendorf AG, Hamburg, Germany). The separation of derivatized extracts (1:1) was performed on a 7890 A gas chromatograph (Agilent, Atlanta, GA, USA) equipped with a 7683B auto-sampler and a 30 m × 0.25-mm i.d. fused silica capillary column with a 0.25-µm CP-SIL 8 CB low bleed coating (Varian Inc., Palo Alto, CA, USA). A 1.0 mL/min discharge of helium gas was present, and the injector’s temperature was 290°C. The temperature program was started at 200°C, held for two minutes, then increased by 10°C per minute to 310°C and kept for ten minutes. A Pegasus HT-TOF mass spectrometer (LECO, St. Joseph, MI, USA) with an m/z range of 50–600 was utilized for sample analysis. Transmission line, detector, and ion source temperatures were set to 280°C, 230°C, and 1800 V, respectively. A calibration curve was generated using twelve lipophilic standards within a weight range of 0.01 to 10.0 grams, with 5α-cholestane as the internal standard (IS).

### 2.5 Quantification of minerals

Calcium, magnesium, phosphorus, potassium, sulfate, copper, iron, manganese, sodium, and zinc concentrations were measured using inductively coupled plasma optical emission spectrometry (Integra XL; GBC Co., Melbourne, Australia), following the procedures specified in the MFDS food code (MFDS, 2019c; Kim et al., 2021).

### 2.6 Statistical analyses

Statistical analysis was performed using SAS Enterprise Guide 7.0. A one-way analysis of variance (ANOVA) was applied to assess differences among onion varieties, and regions. Bonferroni-corrected t-tests were used for mean comparisons, with statistical significance set at P ≤ 0.05. To examine differences across sites and varieties, a mixed-model ANOVA was conducted, where entry was treated as a fixed effect and both location and the location-by-entry interaction as random effects (Herman et al., 2013). Quantitative data for Antioxidant, Proximate, etc. were then analyzed using principal component analysis (PCA) with SIMCA version 13 (Umetrics, Umea, Sweden) to explore group differences within multivariate data. The PCA results included score plots for sample comparison and loading plots illustrating cluster separation. For graphical representation, GraphPad Prism 8.0 (Boston, MA 02110, USA) were used.

## 3 Results

### 3.1 Physiological characteristics of onion varieties (Katamaru and Sunpower) grown in Changnyeong and Muan

**Figure 1:**
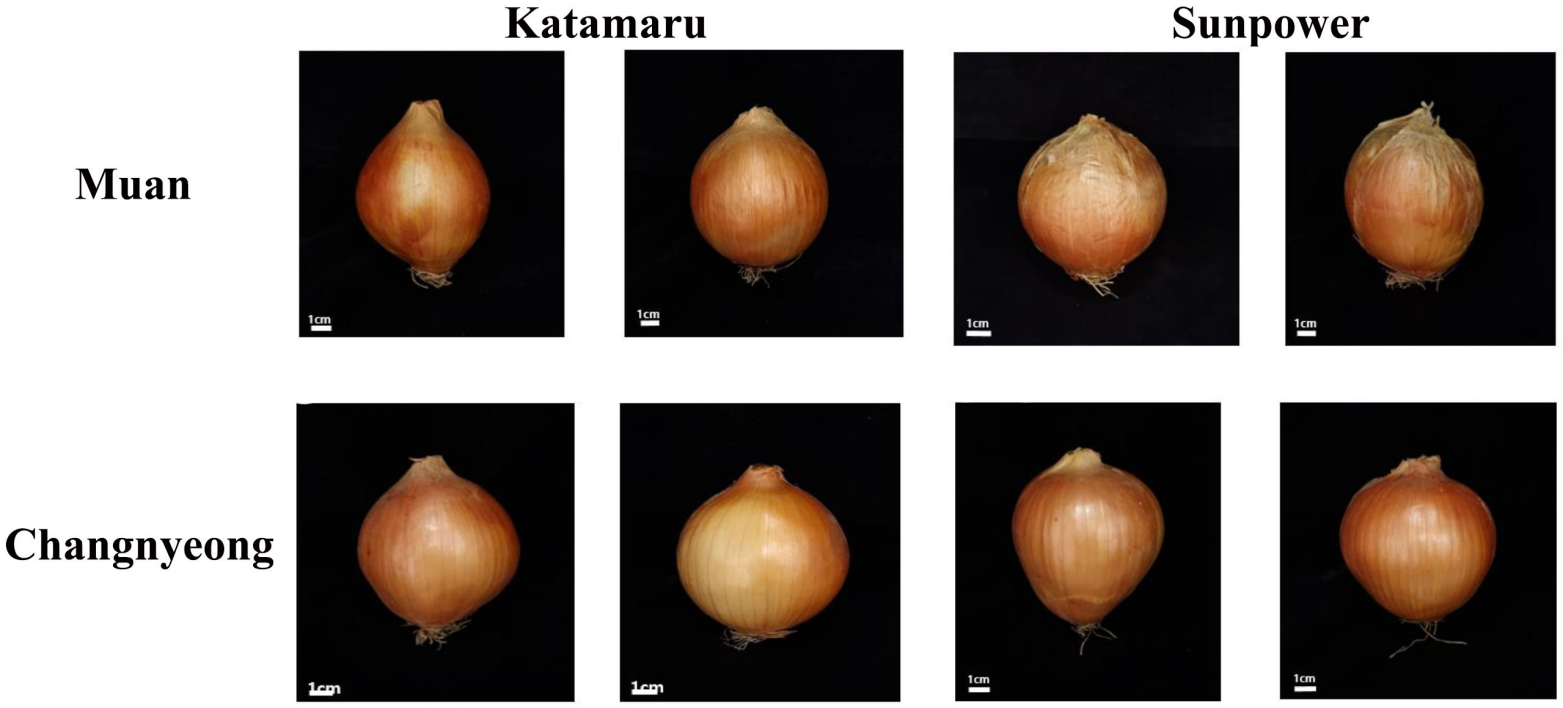
Phenotypic visualization of 2 Onion varieties (Katamaru and sunpower), cultivated at Muan and Changnyeong.

The physiological characteristics of the varieties Katamaru (KM) and Sunpower (SP) grown in Changnyeong and Muan were compared to assess their growth performance. Both varieties showed similar leaf numbers, with no significant difference in leaf numbers between the varieties and locations. However, Katamaru consistently exhibited longer shoot lengths than Sunpower. As in Changnyeong, the Katamaru’s shoot length was 82.9, while Sunpower’s was 76.7cm, and in Muan, Katamaru’s was 81.6 cm compared to Sunpower’s 79.4 cm. This trend was consistent in both locations, indicating that Katamaru tends to grow taller shoots. Similarly, Katamaru showed superior leaf sheet length, with 23.0 cm in Changnyeong and 23.4 cm in Muan, compared to Sunpower 21.1 and 20.8 cm, respectively (**Table 1**, **Figure 1**). This suggests that Katamaru has longer leaves overall. In-contrast, Sunpower had a larger leaf diameter than Katamaru, indicates that Sunpower’s leaves are thicker.

**Table 1:** Phenotypic data of 2 Onion varieties (Katamaru and sunpower), cultivated at Muan and Changnyeong.

Name	Leaf numbers	Shoot length (cm)	Leaf sheet length (cm)	Leaf diameter (mm)	Bulb height (mm)	Bulb width (mm)	Bulb weight (g)	Bulb shape index
Changnyeong								
Katamaru	7.2 ± 0.1a	82.9 ± 4.6a	23.0 ± 2.0a	23.03 ± 1.5a	80.7 ± 3.2a	80.0 ± 4.1b	279.1 ± 4.7b	1.0 ± 0.001a
Sunpower	7.1 ± 0.1a	76.7 ± 5.1b	21.1 ± 2.3b	24.3 ± 1.2a	80.3 ± 2.8a	84.6 ± 3.4a	310.9 ± 5.3a	0.9 ± 0.001b
Muan								
Katamaru	7.1 ± 0.2a	81.6 ± 5.3a	23.4 ± 2.1a	23.3 ± 1.2a	81.2 ± 3.1a	74.5 ± 3.2c	311.4 ± 4.1a	1.0 ± 0.001a
Sunpower	7.0 ± 0.1a	78.4 ± 4.1b	20.8 ± 2.1b	24.9 ± 1.5a	80.5 ± 3.4a	80.6 ± 3.4b	320.5 ± 4.6a	0.9 ± 0.001b
Pvale	0.85	0.041	0.042	0.75	0.88	0.001	0.053	0.043

The data are the means of three replicates ± SEM. Alphabetic letters reflect statistically significant differences (at p<0.05), through two-way ANOVA using SAS Enterprise Guide 7.0.

Regarding bulb characteristics, both varieties showed similar bulb heights, with no significant differences. However, Sunpower consistently had a larger bulb width than Katamaru, Additionally, Sunpower outperformed Katamaru in bulb weight, with 310.9 g and 320.5 g in Changnyeong and Muan, respectively, compared to Katamaru’s 279.1 g and 311.4 g. This suggests that Sunpower produces larger and heavier bulbs overall. Lastly, the bulb shape index was higher for Katamaru, with a value of 1.0 in both locations, indicating a rounder bulb shape compared to Sunpower, which had a bulb shape index of 0.9 in both locations, suggesting a more elongated bulb shape (**Table 1**, **Figure 1**).

### 3.2 Antioxidant activity of two onion varieties across two locations (Muan and Changnyeong)

**Figure 2:**
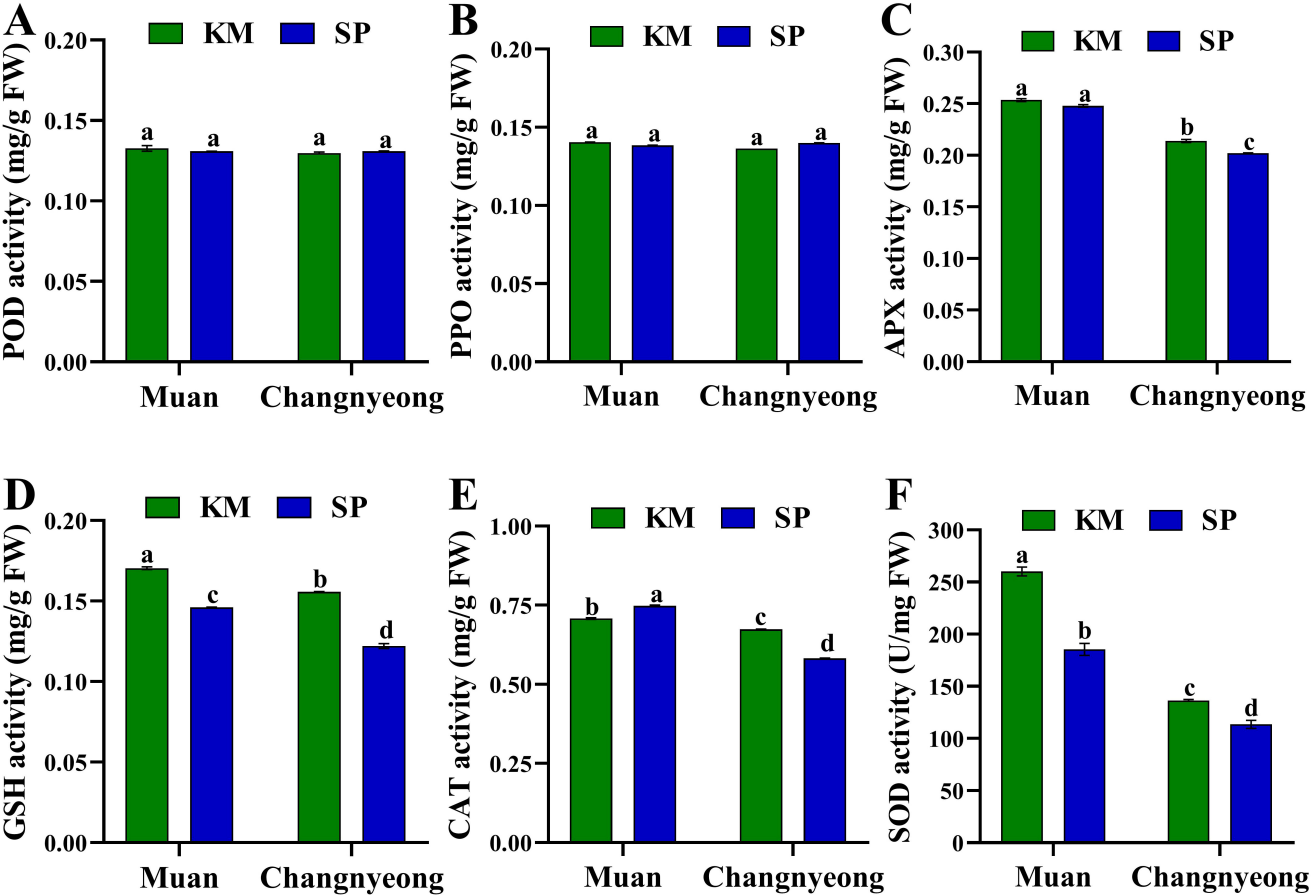
Quantification of antioxidant enzyme activity in two onion varieties Katamaru (KM) and Sunpower (SP) at two locations Muan and Changnyeong. **(A)** POD (peroxidase), **(B)** PPO (polyphenol oxidase), **(C)** APX (ascorbate peroxidase), **(D)** (glutathione), **(E)** CAT (catalase), and **(F)** SOD (superoxide dismutase), The data is the means of three replicates ± SEM. Alphabetical letters on each bar reflect statistically significant difference (at p<0.05), through two way ANOVA using SAS Enterprise Guide 7.0.

The present study shows the activities of various antioxidant enzymes and compounds in two onion varieties, KM and SP, grown in Muan and Changnyeong. The parameters evaluated include activities of POD, PPO, APX, GSH, CAT, and SOD, as well as DPPH radical scavenging activity, total phenolic content (TPC), and total flavonoid content (TFC). Briefly, both KM and SP onions showed comparable POD and PPO activity in Muan and Changnyeong, with no significant differences between varieties and locations (**Figures 2A, B**), indicating that these antioxidant enzymes are less influenced by genotype or environmental factors. On the other hand, APX activity was significantly higher in Muan for both varieties compared to Changnyeong, which may be attributed to Muan’s higher potassium (K 2.16 cmolc/kg) and available phosphorus (P_2_O_5_ 32.03 mg/kg) levels, both of which play crucial roles in oxidative stress regulation. Notably, the KM variety exhibited a greater decline in APX activity in Changnyeong, while SP maintained higher activity than KM in this location (**Figure 2C**). Similarly, GSH activity varied significantly across varieties and locations (**Figure 2D**). KM grown in Muan showed the highest GSH activity, while SP in Changnyeong exhibited the lowest, with KM outperforming SP in both locations. In the case of CAT activity, the KM onions had higher CAT activity in Changnyeong compared to SP, while SP showed better CAT activity in Muan. Despite the variations, both varieties exhibited a noticeable decline in activity when comparing Muan and Changnyeong, with KM maintaining a comparative edge (**Figure 2E**). This trend could be linked to the higher nitrogen (N 0.35%) and organic matter content (OM 53.68 g/kg) in Changnyeong (**Supplementary Table S2**), which may have mitigated oxidative stress, leading to reduced CAT activity. The highest SOD activity was observed in KM onions grown in Muan, followed by SP in Muan. Changnyeong-grown samples showed a marked reduction in SOD activity for both varieties, with SP showing the lowest values (**Figure 2F**). These results show a significant environmental effect, with the climatic differences between the two locations, including Muan’s higher humidity (74-87%) and cooler winter temperatures, may have contributed to the variations in antioxidant enzyme activity. These findings emphasize the interactive effects of soil composition and weather conditions on antioxidant responses in onions, with Muan providing a more favorable environment for oxidative stress-related enzyme activity.

**Figure 3:**
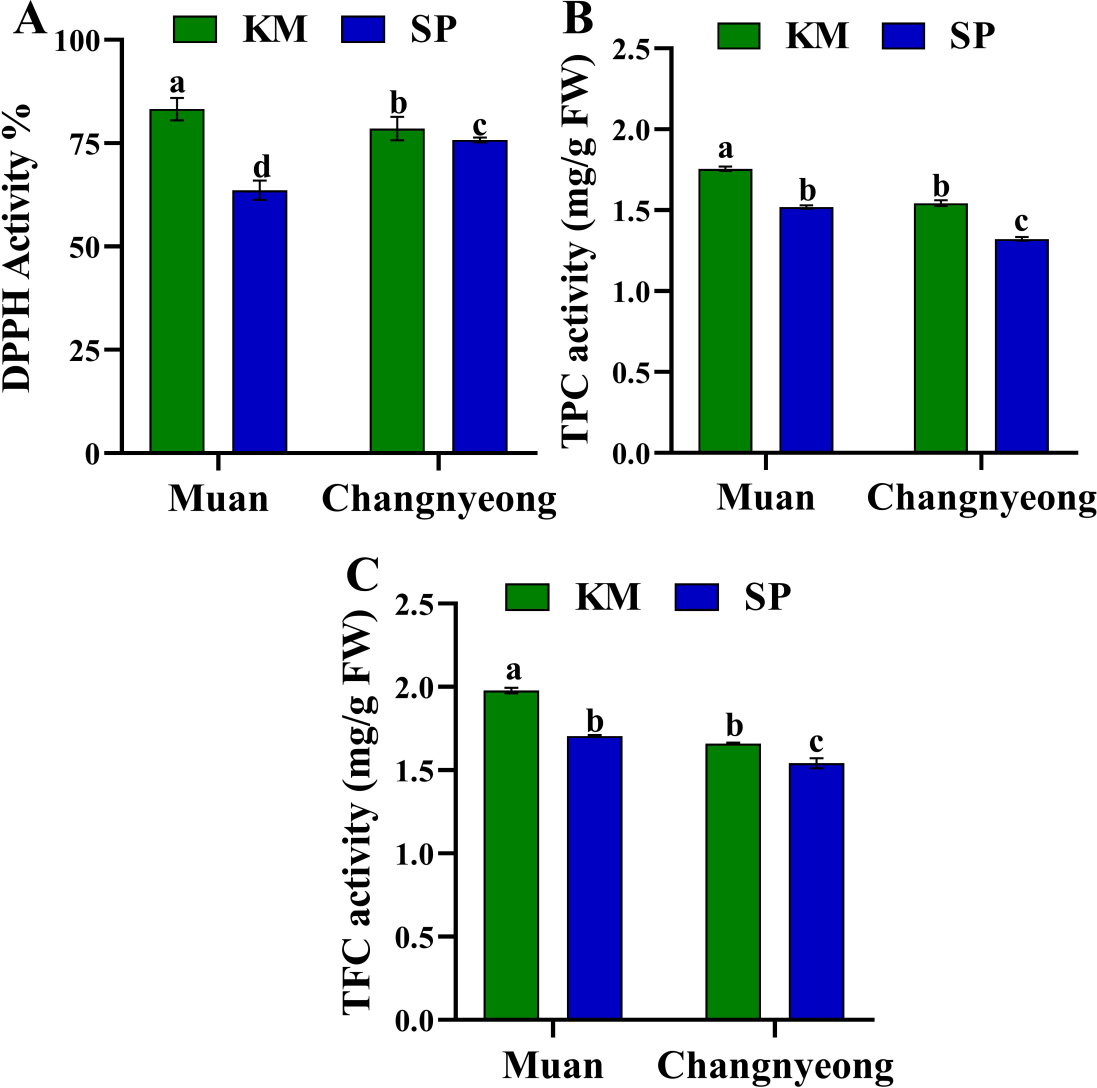
Quantification of antioxidant enzyme activity in two onion varieties Katamaru (KM) and Sunpower (SP) at two location Muan and Changnyeong. **(A)** DPPH activity, **(B)** TPC (total Phenolic content), **(C)** TFC (total flavonoids content). The data is the means of three replicates ± SEM. Alphabetical letters on each bar reflect statistically significant difference (at p<0.05), through two way ANOVA using SAS Enterprise Guide 7.0.

Furthermore, The DPPH activity, was significantly higher in KM onions compared to SP in both locations. Onions grown in Muan exhibited superior DPPH activity across varieties, with KM in Muan having the highest percentage (**Figure 3A**). On the other hand, KM consistently exhibited higher TPC than SP in both locations, with the highest levels recorded in Muan. While the onions grown in Changnyeong showed reduced TPC levels for both varieties, suggesting environmental stress and reduced phenolic synthesis in this location (**Figure 3B**). This aligns with Changnyeong having higher organic matter (53.68 g/kg) and nitrogen content (0.35%), which could influence phenolic biosynthesis by altering nitrogen allocation towards growth rather than secondary metabolite production. Similarly, TFC followed a similar trend as TPC, with KM showing higher levels than SP in both locations. The Muan environment supported higher flavonoid synthesis in both varieties compared to Changnyeong, with KM in Muan having the highest TFC (**Figure 3C**). This may be linked to Muan cooler winter temperatures and higher humidity (74-87%), which could have enhanced stress-induced flavonoid accumulation. Overall, KM onions consistently exhibited superior antioxidant activity and compound content compared to SP across both locations. The more alkaline soil (pH 8.02) and greater phosphorus availability in Muan may have contributed to higher antioxidant enzyme activities, phenolic, and flavonoid synthesis. The results indicate significant genotype × environment interactions, with KM demonstrating greater resilience and adaptability to environmental variations, particularly in response to soil mineral composition and climatic factors.

### 3.3 Proximate composition activity of two onion varieties across two locations (Muan and Changnyeong)

**Figure 4:**
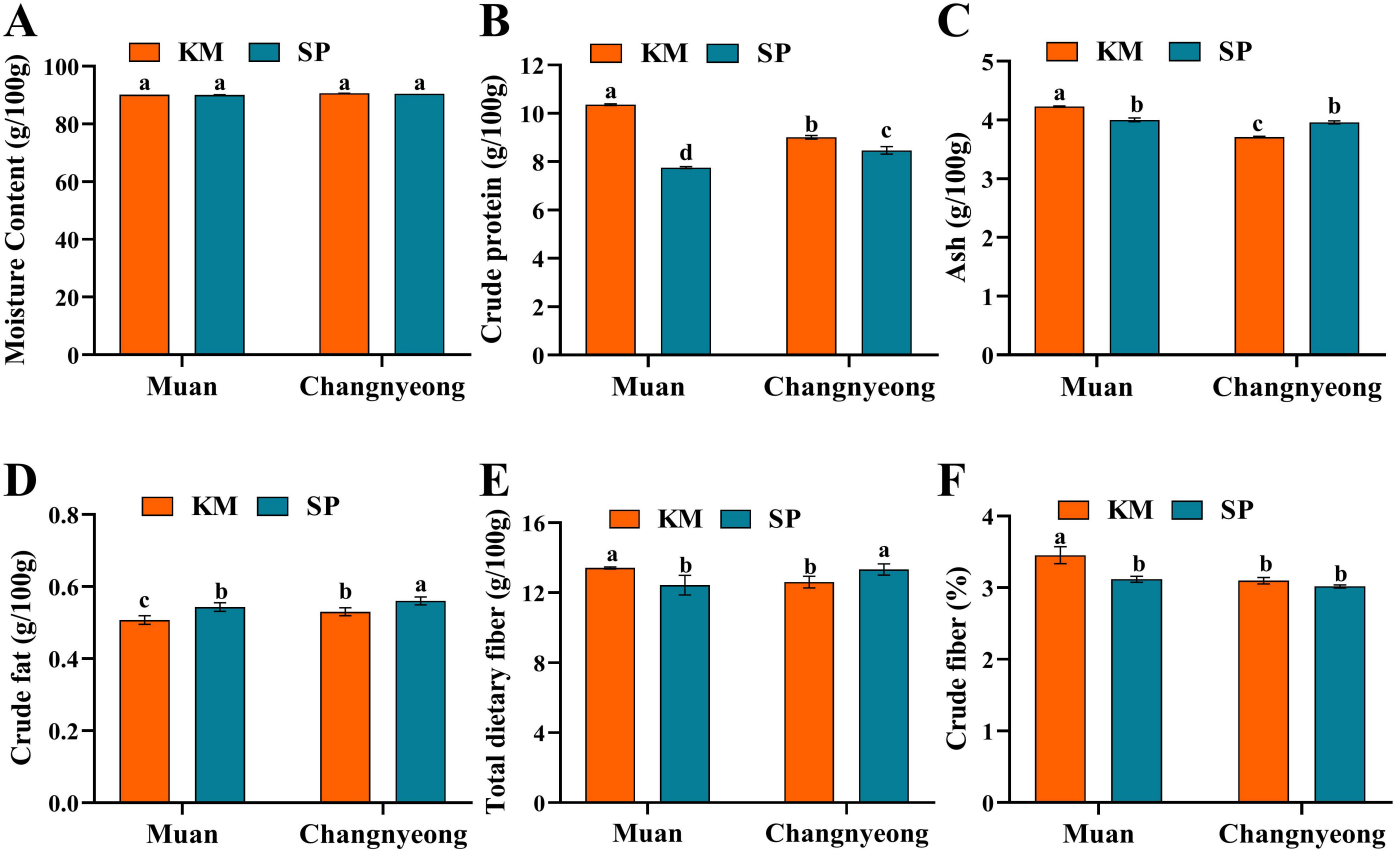
Quantification of proximate component in two onion varieties Katamaru (KM) and Sunpower (SP) at two location Muan and Changnyeong. **(A)** moisture, **(B)** crude protein, **(C)** Ash, **(D)** crude fats, and **(E)** total dietary fiber, and **(F)** crude fiber. The data is the means of three replicates ± SEM. Alphabetical letters on each bar reflect statistically significant difference (at p<0.05), through two way ANOVA using SAS Enterprise Guide 7.0.

The present results shows that both onion varieties, KM and SP, had high moisture content across the two locations, with no significant differences observed between them. This consistency suggests that the environmental factors in Muan and Changnyeong, including humidity levels (Muan 74-87% and Changnyeong 65-82%) and soil moisture retention, did not greatly impact onion moisture levels. The moisture content of KM and SP remained nearly identical at each location. While there was no substantial difference between Muan and Changnyeong in moisture content (**Figure 4A**). The variety KM showed significantly higher protein content than SP at both locations, such as Muan displayed significantly higher crude protein in KM than SP. While in Changnyeong Protein levels for KM decreased slightly compared to Muan but were still higher than SP (**Figure 4B**). Similarly, Ash content was highest for KM grown in Muan, while SP showed slightly lower values. In Changnyeong, ash content decreased slightly compared to Muan for KM and remained stable for SP. Moreover, KM consistently had higher ash content than SP at both locations. Muan onions exhibited higher ash content than those grown in Changnyeong (**Figure 4C**). Since Muan’s soil has higher exchangeable potassium (2.16 cmolc/kg) and available phosphorus, these minerals could have contributed to the elevated ash content observed in onions grown in Muan. In the case of crude fat, KM and SP exhibited differences in crude fat content, with SP having slightly higher levels than KM at both locations, such in Muan the KM shows lower crude fat than SP (**Figure 4D**). This variation could be influenced by the higher organic matter content in Changnyeong (53.68 g/kg and 30.76 g/kg in Muan), which may have facilitated lipid metabolism in onions. On the other hand, KM showed higher dietary fiber than SP across both locations. KM grown in Muan had significantly higher fiber content (13 g/100g) compared to SP. While in Changnyeong both varieties showed a slight decrease in dietary fiber content compared to Muan, but KM remained higher (**Figures 4E, F**). The higher crude fiber content in onions grown in Muan could be associated with higher phosphorus and potassium availability, which are essential for carbohydrate metabolism and cell wall synthesis. Additionally, the cooler early season temperatures in Muan may have contributed to enhanced fiber accumulation, as lower temperatures can promote structural carbohydrate deposition. Overall, the proximate composition analysis highlights significant genotype x environment interactions, with Muan supporting higher protein, ash, and fiber content, while Changnyeong facilitated greater fat accumulation in onions.

### 3.4 Effects on nutrient content in onion varieties across locations (Muan and Changnyeong)

**Figure 5:**
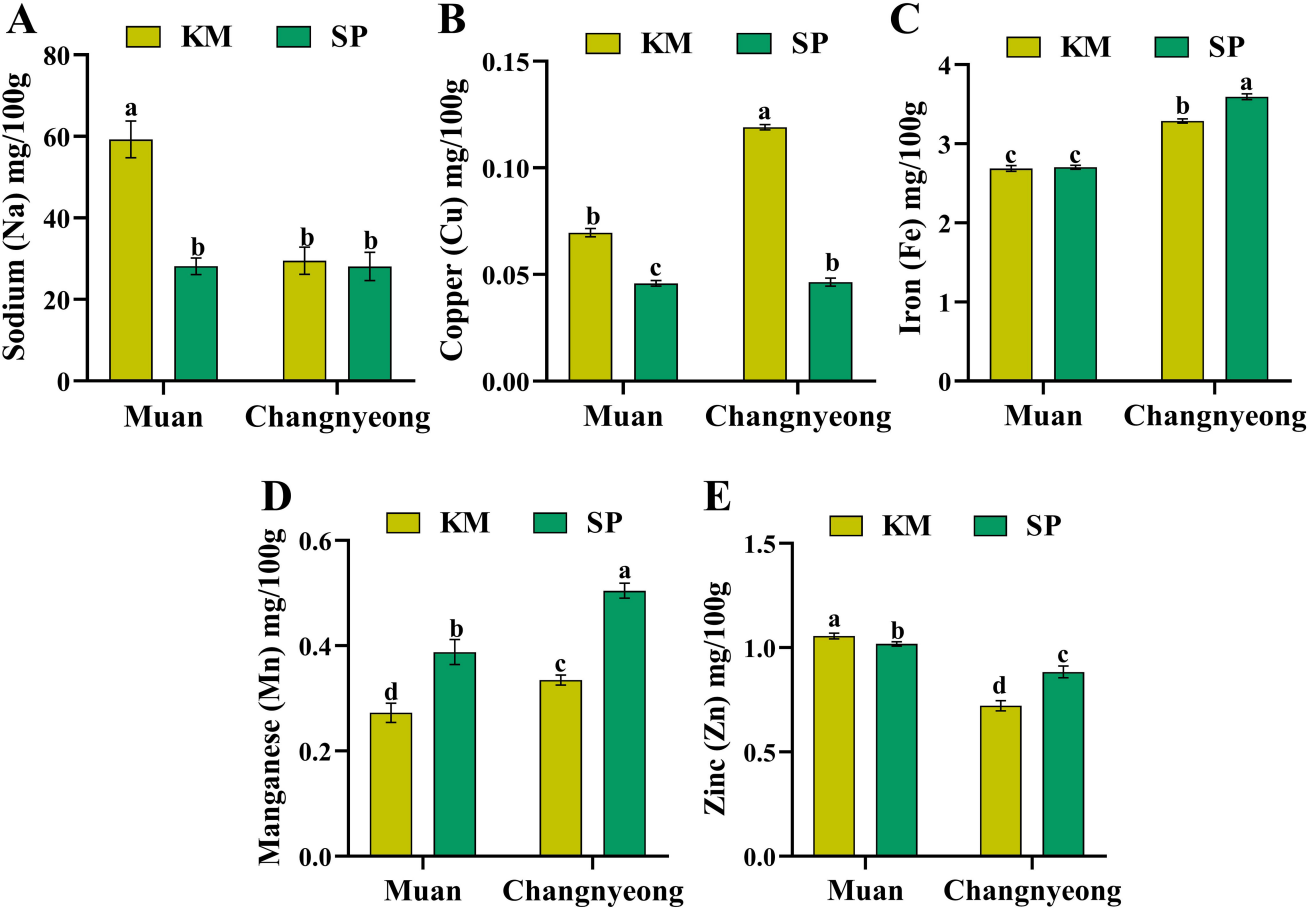
Quantification of macro-nutrients (mg/100 g dry weight) in two onion varieties Katamaru (KM) and Sunpower (SP) at two location Muan and Changnyeong. **(A)** potassium (K), **(B)** phosphate (P), **(C)** calcium (Ca), **(D)** magnesium (Mg), and **(E)** sulfur (S). The data is the means of three replicates ± SEM (n = 3). Alphabetical letters on each bar reflect statistically significant difference at (p<0.05), through two-way ANOVA using SAS Enterprise Guide 7.0.

The concentrations of five essential macronutrients potassium (K), phosphate (P), calcium (Ca), magnesium (Mg), and sulfur (S) in two onion varieties, KM and SP, grown in Muan and Changnyeong. Briefly, KM onions in Changnyeong showed significantly higher potassium content compared to those in Muan (**Figure 5A**). In contrast, SP onions had relatively consistent K levels across both locations. Notably, SP in Changnyeong exhibited the highest potassium content among all samples. Similarly, both varieties displayed higher phosphate content in Muan compared to Changnyeong (**Figure 5B**), which aligns with Muan’s higher available phosphorus levels (Muan 32.03 mg/kg and 24.11 mg/kg in Changnyeong) (**Supplementary Table S2**). KM showed slightly lower P levels than SP in Muan, while SP demonstrated a greater decline in phosphate content when grown in Changnyeong. This highlights that the phosphate content is more affected by location, with Muan being a favorable environment for P accumulation. Calcium content varied significantly across varieties and locations (**Figure 5C**), with SP onions in Changnyeong had the highest calcium concentration, followed by SP in Muan. KM onions showed lower calcium levels in both locations, with a pronounced decrease in Muan. This indicates that the SP variety is better at accumulating calcium, especially in Changnyeong. Magnesium accumulation followed a similar trend, with SP onions consistently had higher magnesium content than KM in both locations (**Figure 5D**). The highest Mg concentration was observed in SP grown in Muan, whereas KM in Changnyeong recorded the lowest, which could be attributed to Muan higher soil exchangeable K levels (2.16 cmolc/kg and 1.31 cmolc/kg in Changnyeong), as potassium and magnesium uptake often show competitive interactions. Sulfur content was generally higher in Muan compared to Changnyeong for both varieties (**Figure 5E**), with SP showed slightly higher S content than KM in both locations. Interestingly, KM in Muan exhibited a significant increase in sulfur accumulation compared to Changnyeong, suggests that sulfur content is more influenced by environmental conditions, particularly Muan’s greater phosphorus availability and slightly cooler seasonal temperatures, which may enhance sulfur assimilation.

**Figure 6:**
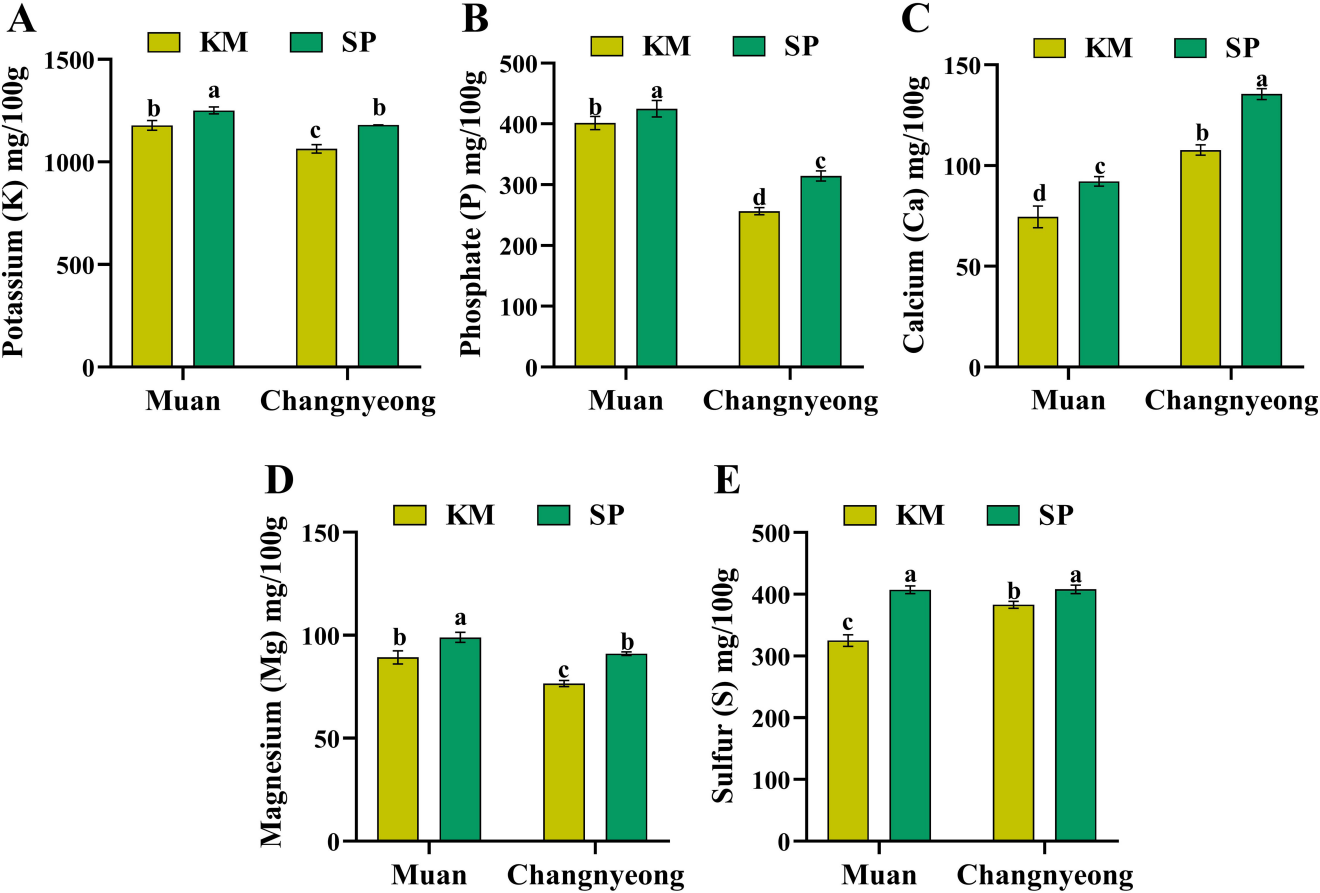
Quantification of micro-nutrients (mg/100 g dry weight) in two onion varieties Katamaru (KM) and Sunpower (SP) at two location Muan and Changnyeong. **(A)** sodium (Na), **(B)** copper (Cu), **(C)** iron (Fe), **(D)** manganese (Mn), and **(E)** zinc (Zn). The data is the means of three replicates ± SEM (n = 3). Alphabetical letters on each bar reflect statistically significant difference (at p<0.05), through two way ANOVA using SAS Enterprise Guide 7.0.

Furthermore, the micronutrients sodium, copper, iron, manganese, and zinc in two onion varieties, KM and SP, grown in Muan and Changnyeong. Briefly, KM onions in Muan showed significantly higher sodium content compared to those in Changnyeong (**Figure 6A**). In contrast, KM and SP in Changnyeong had relatively consistent Na levels across both locations. On the other hand, KM showed higher Cu levels than SP in Changnyeong and Muan, while SP demonstrated a greater decline in Cu content when grown in Changnyeong. This highlights that the Cu content is more affected by variety (**Figure 6B**). Similarly, the SP and KM grown in the Changnyeong shows the highest Fe content then Muan (**Figure 6C**), which may be linked to the region’s slightly lower pH (7.29 and 8.02 in Muan), enhancing Fe solubility and uptake. The SP onions in Changnyeong had the highest Mn concentration, followed by SP in Muan. Where KM onions showed lower Mn levels in both locations, with a pronounced decrease in Muan (**Figure 6D**). Similarly, in Muan the KM and SP exhibited the highest zinc concentration, whereas KM and SP in Changnyeong recorded the lowest (**Figure 6E**), suggesting that Zn availability may be enhanced by Muan higher phosphorus levels, as phosphorus is known to interact with Zn uptake. Overall, SP consistently outperformed KM in terms of nutrient accumulation, particularly for calcium, magnesium, and potassium, indicating its superior nutrient uptake efficiency. Muan emerged as the more favorable location for phosphate and sulfur accumulation, while Changnyeong supported higher potassium and calcium levels, highlighting significant genotype x environment interactions in onion nutrient composition. The macro and micro-nutrient analysis reveals significant variation influenced by both variety and location. SP consistently outperformed KM in terms of nutrient accumulation, especially for calcium, magnesium, and potassium, indicating its superior nutrient uptake efficiency. Muan emerged as the more favorable location for phosphate and sulfur accumulation, while Changnyeong supported higher potassium and calcium levels. This indicates genotype x environment interactions, with SP showing better adaptability to variable environmental conditions.

### 3.5 Amino acid content in two onion varieties across two locations (Muan and Changnyeong)

The analysis of amino acid composition in onion varieties KM and SP across two locations, Muan and Changnyeong were summarized in the current study. Briefly, in Muan the KM exhibited higher amino acid concentrations compared to SP in Muan, except for Glu, Gly, Val, Tyr, and Cys, where the differences were slight. Significant differences were observed in Asp, Thr, and Arg. While in Changnyeong, the KM showed higher levels of most amino acids compared to SP, such as Glu (KM 2.43 and SP 2.26), Leu (KM 0.42 and SP 0.39), and Arg (KM 1.68 and SP 1.28) (**Table 2**). SP presented lower concentrations across most amino acids, with only Cys showing a slight advantage (SP 0.18 and. KM 0.16). These differences can be linked to soil composition, as Changnyeong has higher nitrogen (0.35%) and organic matter content (53.68 g/kg) likely contributed to increased amino acid synthesis, particularly nitrogen rich amino acids such as Glu and Arg. Furthermore, when comparing the locations Muan and Changnyeong, the KM in Muan, the amino acids Asp (0.82), Thr (0.24), and Arg (1.44) were significantly lower than their respective levels in Changnyeong (Asp 0.63, Thr 0.23, Arg 1.68). Where the amino acids Gly (0.18 in Muan vs. 0.22 in Changnyeong) and Val (0.29 in Muan vs. 0.26 in Changnyeong) were higher in Muan. Similarly, SP in Muan, the levels of Asp (0.65), Thr (0.22), and Arg (1.29) were generally lower compared to Changnyeong (Asp 0.60, Thr 0.23, Arg 1.28). Where Changnyeong exhibited higher levels of specific amino acids such as Glu and Leu (**Table 2**). The smaller variations in SP compared to KM across both locations suggest a stronger genetic influence on amino acid composition in SP, whereas KM appears more sensitive to environmental changes. Additionally, the higher humidity (74-87%) and slightly cooler seasonal temperatures in Muan may have enhanced stress induced metabolic adjustments, leading to increased Asp and Thr levels, while Changnyeong warmer temperatures and nutrient rich soil promoted Glu, Gly, and Leu accumulation (**Supplementary Tables S1**, **S2**). Moreover, Muan had higher averages of Asp, Thr, and Arg. Changnyeong showed significantly higher Glu, Gly, and Leu. Overall, KM shows higher amino acid levels than SP across both locations, with Changnyeong favoring amino acid accumulation, particularly for Glu, Gly, and Leu. These findings underscore the importance of both varietal selection and cultivation environment in shaping the amino acid profile of onions, offering valuable insights for optimizing nutritional quality through agricultural management.

**Table 2:** Quantification of amino acid in two onion varieties Katamaru (KM) and Sunpower (SP) at two cultivation location Muan and Changnyeong.

Name	Asp	Thr	Ser	Glu	Gly	Ala	Val	Ile	Leu	Tyr	Phe	Lys	His	Arg	Pro	Trp	Cys	Met
Muan																		
KM	0.82 ± 0.02a	0.24 ± 0.01a	0.33 ± 0.0a	2.34 ± 0.05ab	0.18 ± 0.01b	0.28 ± 0.01a	0.29 ± 0.01a	0.20 ± 0.01a	0.37 ± 0.01c	0.24 ± 0.01a	0.29 ± 0.01b	0.45 ± 0.02a	0.17 ± 0.0a	1.44 ± 0.06b	0.10 ± 0.0a	0.04 ± 0.0a	0.14 ± 0.0c	0.06 ± 0.0a
SP	0.65 ± 0.03b	0.22 ± 0.01b	0.32 ± 0.01a	2.09 ± 0.02c	0.17 ± 0.0b	0.27 ± 0.0a	0.25 ± 0.0b	0.17 ± 0.0b	0.34 ± 0.01d	0.21 ± 0.01b	0.28 ± 0.01b	0.41 ± 0.01b	0.13 ± 0.0c	1.29 ± 0.02c	0.08 ± 0.0b	0.04 ± 0.0a	0.15 ± 0.01bc	0.05 ± 0.0a
Changnyeong																		
KM	0.63 ± 0.02b	0.23 ± 0.01ab	0.33 ± 0.01a	2.43 ± 0.03a	0.22 ± 0.01a	0.23 ± 0.01b	0.26 ± 0.01b	0.19 ± 0.0a	0.42 ± 0.01a	0.22 ± 0.01b	0.35 ± 0.01a	0.45 ± 0.01a	0.15 ± 0.00b	1.68 ± 0.03a	0.10 ± 0.00a	0.04 ± 0.0a	0.16 ± 0.01b	0.05 ± 0.0a
SP	0.60 ± 0.02b	0.23 ± 0.01ab	0.31 ± 0.01a	2.26 ± 0.03b	0.22 ± 0.01a	0.20 ± 0.01c	0.22 ± 0.01c	0.14 ± 0.0c	0.39 ± 0.02b	0.18 ± 0.0c	0.28 ± 0.01b	0.37 ± 0.01c	0.14 ± 0.0bc	1.28 ± 0.02c	0.10 ± 0.0a	0.04 ± 0.0a	0.18 ± 0.01a	0.05 ± 0.0a
*P-value*	**	*	Ns	***	**	***	***	***	***	**	*	**	**	**	Ns	Ns	**	ns
Location																		
Muan	0.79 ± 0.11a	0.23 ± 0.01a	0.32 ± 0.01a	2.21 ± 0.03b	0.17 ± 0.0b	0.27 ± 0.0a	0.26 ± 0.01a	0.18 ± 0.01a	0.35 ± 0.02b	0.22 ± 0.01a	0.28 ± 0.01b	0.43 ± 0.02a	0.15 ± 0.01a	1.26 ± 0.05b	0.08 ± 0.0a	0.04 ± 0.0a	0.14 ± 0.0b	0.05 ± 0.0a
Changnyeong	0.6 ± 0.01b	0.23 ± 0.01a	0.31 ± 0.01a	2.34 ± 0.04a	0.21 ± 0.01a	0.21 ± 0.01b	0.23 ± 0.01	0.16 ± 0.0b	0.40 ± 0.03a	0.19 ± 0.0b	0.31 ± 0.01a	0.40 ± 0.02	0.14 ± 0.0a	1.48 ± 0.06a	0.09 ± 0.0a	0.03 ± 0.0a	0.17 ± 0.01a	0.05 ± 0.0a
*P-value*	**	Ns	Ns	***	***	***	***	**	***	***	***	**	Ns	***	Ns	Ns	**	Ns

The data are the means of three replicates ± SEM. Alphabetical letters on each bar reflect statistically significant difference (at p<0.05), through two way ANOVA using SAS Enterprise Guide 7.0. The Asterisk represents the statistically significant at (*P<0.05), **(P<0.01), and ***(P<0.001), where the Ns represent no significant difference.

### 3.6 PCA-based assessment of compositional diversity of onion varieties across different cultivation locations

**Figure 7:**
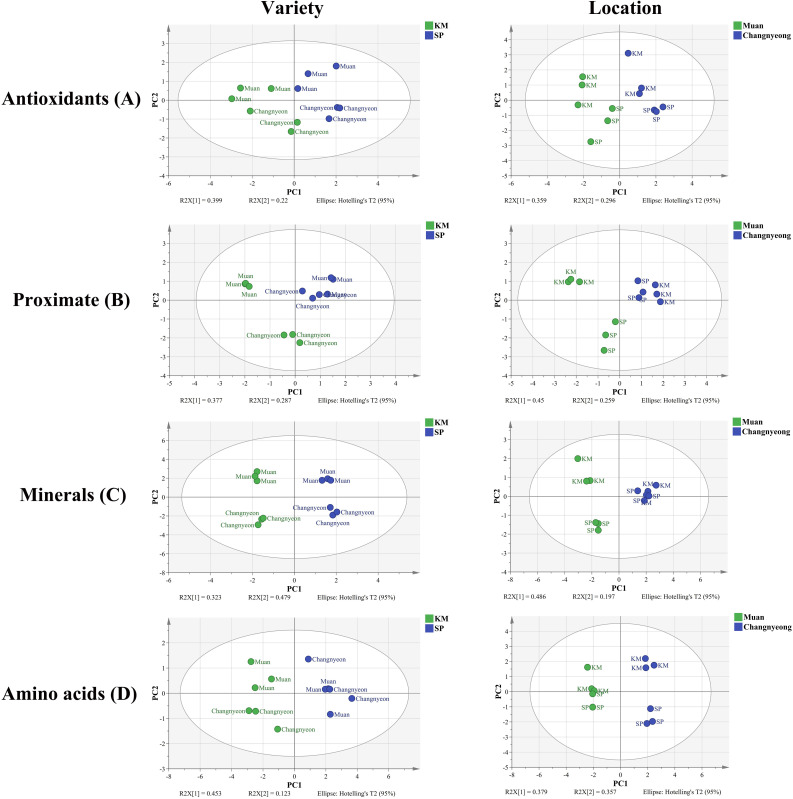
Principal component analysis (PCA), score plots of compositional data of two onion varieties Katamaru (KM) and Sunpower (SP) at two location Muan and Changnyeong. The **(A)** Antioxidants, **(B)** proximate, **(C)** minerals, and **(D)** amino acid data were subjected to PCA by variety and location. The PCA model was obtained with five principal components to explain the variance among samples grouped by variety and location.

The principal component analysis (PCA) revealed distinct compositional differences between the two onion varieties, Katamaru (KM) and Sunpower (SP), cultivated in two different locations, Muan and Changnyeong. Across all compositional amino acids, minerals, proximate components, and antioxidants, a clear clustering patterns were observed, showing the influence of both genotype and cultivation environment on onion composition. For amino acids, KM and SP exhibited distinct separation (PC1 45.3%, PC2 21.3%), with KM samples clustering in one region and SP samples in another, indicating significant varietal differences at p value< 0.02. Additionally, onions cultivated in Muan were positioned differently from those in Changnyeong, suggesting that location strongly influenced amino acid accumulation (**Figure 7**). The wider spread of onion samples grown in Muan suggests greater compositional variation in this location compared to Changnyeong. A similar trend was observed for minerals (PC1 33.3%, PC2 17.9%), where KM and SP formed distinct clusters, reinforcing the role of genetic factors in mineral composition. The effect of location was also evident, with Muan and Changnyeong samples forming separate clusters, especially for KM, which displayed greater dispersion, indicating a stronger environmental influence, which showed significant location effects (p<0.01) for elements such as potassium, magnesium, and calcium. The proximate composition (PC1 37.7%, PC2 27.1%) followed a comparable trend, with KM and SP forming distinct clusters, demonstrating varietal specificity in moisture, protein, and carbohydrate content. Interestingly, SP samples exhibited a more compact distribution across both locations, suggesting a relatively stable composition irrespective of environmental conditions. In contrast, KM samples were more dispersed, particularly in Muan, reflecting a greater environmental influence on its proximate composition (p value<0.03). Antioxidant composition further supported this pattern (PC1 39.0%, PC2 22.2%), with KM and SP separated, confirming that varietal differences dictate antioxidant profiles. Additionally, samples from Muan showed wider variability in antioxidant levels compared to those from Changnyeong (p value<0.002), showing that environmental conditions in Muan may enhance compositional diversity. Comparing the two varieties, KM consistently exhibited greater variability across all compositional traits, indicating that this variety is more responsive to environmental fluctuations, whereas SP maintained a more stable profile. When comparing locations, Muan grown samples generally showed greater dispersion in PCA plots, suggesting a higher influence of environmental factors on compositional traits in this region compared to Changnyeong (**Figure 7**). This pattern was especially significant for KM, highlighting a strong genotype and environment interaction where KM was more susceptible to location specific influences, while SP remained relatively stable across different cultivation sites. Overall, the PCA results demonstrate that both variety and location significantly influence onion composition, with KM showing a more dynamic response to environmental conditions, while SP maintains a more consistent compositional profile. The distinct clustering patterns for each variety within each location suggest a complex interplay between genetic and environmental factors, contributing to the observed compositional diversity in onions.

## 4 Discussion

Onions (*Allium cepa* L.) are recognized for their rich phytochemical composition, particularly their antioxidant properties. The presence of bioactive compounds such as flavonoids, organosulfur compounds, and phenolics contributes significantly to their health benefits, including anti-inflammatory, anticancer, and cardioprotective effects. The differences in growth performance and physiological traits observed between Katamaru and Sunpower suggest potential genotype-by-environment interactions that may influence both yield and quality-related traits. Understanding these varietal responses across different growing conditions is essential for optimizing onion production and selecting cultivars suited to specific environments. Therefore, present study focused on evaluating the comparative analysis of two onion varieties (Katamaru (KM) and Sunpower (SP)) cultivated in two different locations (Muan and Changnyeong) in Korea.

Our findings revealed that the morphological differences between Katamaru and Sunpower varieties remained consistent across both growing locations, suggesting strong genetic rather than environmental influence (**Figure 1**; **Table 1**). Katamaru’ssuperior shoot length and leaf sheet length aligns with findings by Lee et al. (2020), who reported that some varieties allocate more resources to vegetative growth such as in current study Sunpower’s larger bulb dimensions and weight also support his observations that certain varieties are genetically predisposed to greater bulb development (Lee et al., 2020). The consistent bulb shape index (rounder for Katamaru, more elongated for Sunpower) corroborates Khokhar (2019) conclusion that bulb morphology is primarily genetically determined (Khokhar, 2019). The consistency of varietal differences across both Changnyeong and Muan indicates strong genotypic stability, a characteristic highlighted by Petropoulos et al. (2016) as crucial for commercial cultivars (Petropoulos et al., 2016). Similarly, antioxidants are known to have beneficial effects in plant response to environmental changes. Studies have comparing the antioxidant activity of onions bulbs and their byproduct in many parts of onion (Zhang et al., 2016; Ola-Mudathir et al., 2018). Our findings revealed substantial variations in enzymatic and non-enzymatic antioxidant activities among these varieties and locations, emphasizing the influence of genetic and environmental factors. Plant antioxidant systems comprise several key enzymes, including POD, PPO, APX, GSH, CAT and SOD. In this study, GSH activities were significantly higher in KM onions grown in Muan and Changnyeong compared to SP, with KM consistently outperforming SP (**Figure 2**). These findings align with those of (Kim et al., 2022), who reported similar variations in antioxidant enzyme activities among onion varieties. Additionally, higher potassium (K 2.16 cmolc/kg) and phosphorus (P_2_O_5_ 32.03 mg/kg) levels in Muan likely contributed to enhanced oxidative stress mitigation, consistent with previous reports emphasizing the role of soil mineral composition in antioxidant responses (Sagar et al., 2022; Kim et al., 2024). The relationship between soil mineral composition and antioxidant enzyme activity has been further elucidated by Ibrahim et al. (2023), who demonstrated that potassium supplementation significantly enhanced APX and GSH activities in Allium species under various environmental conditions (Ibrahim et al., 2023). This trend is also in agreement with findings by (Pareek et al., 2017), who demonstrated that onion varieties with higher phenolic content exhibit greater antioxidant potential. The variation in antioxidant activity observed between KM and SP further supports previous studies that suggest that genotype plays a crucial role in determining the overall phytochemical composition of onions (Bibi et al., 2022; Kumar et al., 2024). The results highlight significant genotype x environment interactions. Muan’s more alkaline soil (pH 8.02) and higher phosphorus content appeared to favor antioxidant enzyme activity and phenolic biosynthesis. This is consistent with studies by (Marrelli et al., 2018), who suggested that soil pH and nutrient availability significantly impact secondary metabolite accumulation in Allium species. Soil conditions and environmental stressors are known to significantly influence the biosynthesis of flavonoids and other antioxidants in onion varieties, further reinforcing the trends observed in this study (Shukla et al., 2023).

Furthermore, the present study revealed significant genotype x environment interactions in the proximate composition of onions grown in Muan and Changnyeong. Both onion varieties, KM and SP, exhibited similar moisture content across the two locations, indicating that environmental factors, such as humidity and soil moisture, had minimal impact on moisture levels. This in line with previous research, which found that moisture content in onions remains relatively stable across diverse climates, as long as adequate soil moisture is maintained (Fatideh and Asil, 2012). In terms of protein content, KM consistently outperformed SP at both locations, with higher protein levels in Muan. This difference can likely be attributed to Muan higher phosphorus availability (32.03 mg/kg and 24.11 mg/kg in Changnyeong), which supports protein biosynthesis (McCallum et al., 2001). Although KM protein content decreased slightly in Changnyeong, it still remained higher than that of SP, suggesting a strong genotype effect. Ash content followed a similar pattern, with KM consistently having higher ash levels than SP (**Figure 4**). In contrast, the higher organic matter content in Changnyeong (53.68 g/kg and 30.76 g/kg in Muan) may have facilitated lipid metabolism, resulting in slightly higher crude fat content in SP compared to KM (Jamir et al., 2013). Previous studies have linked high phosphorus and potassium availability with increased fiber accumulation in crop (Salem et al., 2023). This finding is consistent with other studies suggesting that soil organic matter can enhance the accumulation of lipids in plants.

Additionally, potassium (K), phosphorus (P), calcium (Ca), magnesium (Mg), and sulfur (S) were quantified across both varieties and locations. SP onions exhibited higher Ca and Mg levels compared to KM, whereas KM demonstrated superior phosphate and sulfur accumulation. This finding aligns with studies by (Sagar et al., 2022), which reported varietal differences in macronutrient uptake efficiency influencing overall plant metabolism. Regarding trace elements, KM onions in Muan displayed significantly higher sodium (Na) and copper (Cu) content than those in Changnyeong (**Figures 5**, **6**). Interestingly, iron (Fe) content was higher in Changnyeong-grown samples, which is in agreement with (Park, 2012), who suggested that lower soil pH enhances Fe solubility and plant uptake. A more recent study by Hernández-Apaolaza et al. (2020) has provided mechanistic insights into this relationship, demonstrating that soil pH below 7.5 significantly enhances iron bioavailability through increased dissolution of Fe(III) oxides (Pareek et al., 2017; Hernández-Apaolaza et al., 2020; Elghamry et al., 2024). Moreover, Zn interaction has been further characterized by Nadeem et al. (2024), who demonstrated that moderate phosphorus levels optimize zinc uptake in *Allium cepa*, whereas excessive P can induce Zn deficiency (Nadeem et al., 2024). On the other hand, amino acid quantification revealed that KM consistently exhibited higher levels than SP, with significant variations between locations. Changnyeong-grown onions displayed increased glutamate (Glu), glycine (Gly), and leucine (Leu) (**Table 2**), likely due to the region’s higher nitrogen content. These results are comparable to those reported by (Canton, 2021), which found that nitrogen availability plays a crucial role in amino acid biosynthesis in onion cultivars. Additionally, (Zhao et al., 2021) demonstrated that amino acid profiles in Allium species are strongly influenced by the interaction between genotype and soil nitrogen availability, which supports our observations regarding KM and SP varieties. Similarly, amino acid variations have been linked to soil conditions and climate factors in other studies, reinforcing the role of environmental adaptation in onion metabolism (Wang et al., 1996; El-Abagy et al., 2014). Similarly, recent metabolomic analyses by Ren et al. (2024) have expanded our understanding of nitrogen metabolism in onions, revealing that soil nitrogen content directly modulates the expression of key genes involved in amino acid biosynthesis pathways (Lone et al., 2015; Sharma and Lee, 2016; Krähmer et al., 2021; Ren et al., 2024). Overall, our findings underscore the complex interplay between genetic factors and environmental conditions in determining the component composition of onions (**Figure 7**). These results are in agreement with previous studies that highlight how both cultivar selection and cultivation environment can significantly influence the nutritional and flavor profiles of onion bulbs. This study provides comprehensive insights into the antioxidant activity, nutrient composition, and metabolic variations of two onion varieties grown in different environments. The results indicate that KM consistently exhibits superior antioxidant properties compared to SP, with Muan providing a more favorable environment for enzymatic and non-enzymatic antioxidant responses. The significant genotype x environment interactions observed highlight the need for targeted breeding and cultivation strategies to enhance onion quality. Future research should explore the molecular mechanisms underlying these variations, with a focus on optimizing cultivation practices to maximize health-beneficial compounds in onions.

## 5 Conclusion

This study provides a comprehensive analysis of the antioxidant activity, phytochemical composition, and nutrient variations in two onion varieties grown under different environmental conditions. The findings indicate that genotype and environmental factors play a crucial role in determining the antioxidant potential and nutrient accumulation in onions. KM demonstrated higher antioxidant enzyme activities and non-enzymatic antioxidant content compared to SP, with Muan-grown onions outperforming those cultivated in Changnyeong. The differences in soil pH, phosphorus availability, and organic matter content significantly influenced antioxidant activity and nutrient uptake. Additionally, the variation in minerals, proximate and amino acids content between the two locations highlights the influence of environmental factors. The study underscores the importance of selecting the appropriate onion variety and cultivation site to enhance nutritional and functional properties. Future research should focus on the molecular mechanisms underlying these genotype x environment interactions, particularly the regulation of antioxidant pathways and nutrient transport genes. Targeted breeding programs could focus on enhancing antioxidant enzyme expression and nutrient efficiency in KM-like cultivars. Furthermore, site specific soil management strategies such as phosphorus amendment and pH adjustment could be optimized to improve onion quality. The results of this study contribute valuable knowledge for sustainable crop improvement, agricultural decision-making, and the development of functional food that promoting health through utilization of onions as a natural source of antioxidants and essential nutrients.

## Data Availability

The original contributions presented in the study are included in the article/**Supplementary Material**. Further inquiries can be directed to the corresponding author.
