# Correction: Environmental and genetic effects on phytochemical and nutritional composition of onion (*Allium cepa* L.) varieties in South Korea

**DOI:** 10.3389/fpls.2025.1690012

**Published:** 2025-09-02

**Authors:** Muhammad Imran, Hajeong Kang, Eun-Ha Kim, Sang-Gu Lee, Hyun-Min Park, Hanyoung Choi, Seong-Hoon Kim, Seonwoo Lee, Seonwoo Oh

**Affiliations:** ^1^ Department of Applied Biosciences, Kyungpook National University, Daegu, Republic of Korea; ^2^ Biosafety Division, National Institute of Agriculture Science, Rural Development Administration, Jeonju, Republic of Korea; ^3^ National Agrobiodiversity Center, National Institute of Agriculture Science, Rural Development Administration, Jeonju, Republic of Korea; ^4^ Department of Statistics, Dongguk University, Seoul, Republic of Korea

**Keywords:** onion (*Allium cepa*), antioxidant activity, phytochemical composition, genotype × environment interaction, soil nutrients, environmental adaptation

Author “Seong-Hoon Kim” was erroneously spelled as “Sung Hoon Kim”.

The original version of this article has been updated.

