# Supplementary material for: Environmental and genetic effects on phytochemical and nutritional composition of onion (Allium cepa L.) varieties in South Korea
Source: Front Plant Sci. 2025 Aug 15;16:1649912. doi: 10.3389/fpls.2025.1649912 (PMC12395573; doi:10.3389/fpls.2025.1649912)
Supplement: Supplementary file 1 [file Table1.docx]

Supplementary Material

**Environmental and Genetic Effects on Phytochemical and Nutritional Composition of Onion (Allium cepa L.) Varieties in South Korea**

Muhammad Imran^1,2^, Hajeong Kang^2^, Eun-Ha Kim^2^, Sang-Gu Lee^2^, Hyun-Min Park^2^, Hanyoung Choi^2^, Sung-Hoon Kim^3^, Seon-Woo Lee^4^, Seon-woo Oh^1*^

^1^Department of Applied Biosciences, Kyungpook National University, Daegu 41566, South Korea

^2^Biosafety Division, National Institute of Agriculture Science, Rural Development Administration, Jeonju 54874, Republic of Korea

^3^National Agrobiodiversity Center, National Institute of Agriculture Science, Rural Development Administration, Jeonju 54874, Republic of Korea

^4^Department of Statistics, Dongguk University, Seoul, 04620, Republic of Korea

*Correspondence: [ohsw0507@korea.kr](mailto:ohsw0507@korea.kr);

Tel.: +82-63-238-4708

**Table S1.1. Climatic information of Changnyeong from November 2023 to June 2024**

| **Month** | **Avg High Temp (°C)** | **Avg Low Temp (°C)** | **Avg Temp (°C)** | **Avg Rainfall (mm)** | **Avg Humidity (%)** |
| --- | --- | --- | --- | --- | --- |
| **November** | 15.8 | 4.8 | 10.3 | 52.3 | 68 |
| **December** | 10.1 | 0.1 | 5.1 | 25.8 | 68 |
| **January** | 7.6 | -2.3 | 2.7 | 23.6 | 67 |
| **February** | 10.1 | -0.8 | 4.7 | 35.1 | 66 |
| **March** | 14.8 | 3.6 | 9.2 | 52.3 | 65 |
| **April** | 20.8 | 9.1 | 14.9 | 90.4 | 66 |
| **May** | 25.8 | 14.3 | 20.1 | 101.6 | 70 |
| **June** | 28.9 | 20.1 | 24.5 | 177.8 | 77 |

Source: Agricultural Weather Information Service (http://weather.rda.go.kr)

^a^ Minimum, maximum, and average value of daily temperature

^b^ Total volume of rainfall during one month

**Table S1.2. Climatic information of Muan from November 2023 to June 2024**

| **Month** | **Avg High Temp (°C)** | **Avg Low Temp (°C)** | **Avg Temp (°C)** | **Avg Rainfall (mm)** | **Avg Humidity (%)** |
| --- | --- | --- | --- | --- | --- |
| **November** | 14.0 | 6.7 | 10.3 | 65.8 | 74 |
| **December** | 7.2 | 0.0 | 3.6 | 55.6 | 74 |
| **January** | 5.0 | -1.7 | 1.7 | 43.7 | 75 |
| **February** | 6.7 | -0.6 | 3.1 | 44.2 | 73 |
| **March** | 11.7 | 2.8 | 7.2 | 88.4 | 73 |
| **April** | 16.7 | 8.3 | 12.5 | 104.9 | 72 |
| **May** | 22.2 | 13.3 | 17.8 | 103.1 | 75 |
| **June** | 25.6 | 18.9 | 22.2 | 117.6 | 82 |

Source: Agricultural Weather Information Service (http://weather.rda.go.kr)

^a^ Minimum, maximum, and average value of daily temperature

^b^ Total volume of rainfall during one month

**Table S2.** Soil component analysis of Muan and Changnyeong.

|  | **Muan** | **Changnyeong** |
| --- | --- | --- |
| pH (1:5) | 8.02±0.07a | 7.29±0.09b |
| Electric conductivity (EC) (dS/m) | 0.91±0.01b | 1.59±0.09a |
| Total nitrogen (%) | 0.17±0.01b | 0.35±0.005a |
| OM (g/kg) Organic matter | 30.76±1.6b | 53.68±1.5a |
| K (cmol/kg) Exchangeable potassium | 2.16±0.08a | 1.31±0.02b |
| Ca (cmol/kg) Exchangeable calcium | 9.14±0.4b | 12.20±0.2a |
| Mg (cmol/kg) Exchangeable magnesium | 2.82±0.1b | 3.51±0.03a |
| Na (cmol/kg) Exchangeable natrium | 0.17±0.007b | 0.25±0.009a |
| P205 (mg/kg) Available phosphate | 32.03±1.8a | 24.11±2.6b |

(cmol/kg) represent the centimoles per kilogram, (dS/m) represent Decisimens per meter
